# Observational database study on preeclampsia and postpartum medical care up to 7.5 years after birth

**DOI:** 10.1038/s41598-022-25596-2

**Published:** 2022-12-08

**Authors:** Anna S. Scholz, Kathrin Hassdenteufel, Raphael Gutsfeld, Mitho Müller, Maren Goetz, Armin Bauer, Markus Wallwiener, Sara Y. Brucker, Stefanie Joos, Miriam Giovanna Colombo, Sabine Hawighorst‑Knapstein, Ariane Chaudhuri, Frauke Beck, Stephanie Wallwiener

**Affiliations:** 1 Department of Gynecology and Obstetrics, Heidelberg University Hospital, Im Neuenheimer Feld 440, 69120 Heidelberg, Germany; 2 Department of Psychology, Ludwig Maximilian University, Munich, Germany; 3 Department of General Pediatrics, University Children’s Hospital, Heidelberg, Germany; 4 Department of Women’s Health, University Hospital Tuebingen, Tuebingen, Germany; 5 Institute for General Practice and Interprofessional Care, University Hospital Tuebingen, Tuebingen, Germany; 6 Department of Health Promotion, AOK Baden-Wuerttemberg, Stuttgart, Germany

**Keywords:** Cardiovascular diseases, Hypertension, Pre-eclampsia, Health policy

## Abstract

Preeclampsia is associated with a substantially increased long-term risk for cardiovascular, cerebrovascular and renal disease. It remains unclear whether and to which extent specialized medical postpartum care is sought. We aimed to assess current utilization of postpartum primary and specialized care and medication prescription behavior in women who experienced preeclampsia. This retrospective observational study based on statutory claims data included 193,205 women with 258,344 singleton live births between 2010 and 2017 in Southern Germany. Postpartum care was evaluated by analyzing and comparing the frequency of medical consultations in primary and specialized care and prescriptions for antihypertensive medication among women with and without preeclampsia up to 7.5 years after delivery. Gynecologists and general practitioners were the main health care providers for all women. Although specialized postpartum care was sought by more women after preeclampsia, the effect size indices revealed no considerable association between a history of preeclampsia and the utilization of specialized outpatient aftercare (e.g. 2% vs. 0.6% of patients with and without preeclampsia who consulted a nephrologist during the first year postpartum, r = 0.042). Preeclampsia was associated with an increased risk to take any antihypertensive medication after delivery (HR 2.7 [2.6; 2.8]). Postpartum referral to specialized outpatient care and quarterly prescriptions of antihypertensives following preeclampsia failed to match the early and rapidly increased incidence and risk of hypertension. These data highlight the missed opportunity to implement a reasonable follow-up strategy and prevention management in order to achieve long-term clinical benefits.

Preeclampsia (PE) is a multi-organ disorder characterized by hypertension in the second half of pregnancy combined with features of multi-organ dysfunction^1^. PE affects up to 8% of pregnancies and substantially contributes to maternal and perinatal mortality and morbidity^2,3^. In addition to the short-term complications, evidence of long-term maternal and offspring morbidity after PE is constantly rising. The risk for developing any manifestation of cardiovascular disease (CVD), such as myocardial infarction or ischemic heart disease, at least doubles after PE^4–6^ and increases cardiovascular mortality^7^. Cerebrovascular complications following PE include stroke and Alzheimer disease^8,9^. PE and renal diseases are closely linked as both entities represent a risk factor and complication of the other^10–12^. Moreover, the risk for both chronic kidney disease (CKD) and cardiovascular morbidity is even more considerable for women with recurrent episodes of PE^4^.

Consequently, several international guidelines such as the 2011 American Heart Association’s guideline^13^ and the 2016 European Society of Cardiology (ESC) guideline on cardiovascular risk prevention^2^ acknowledge preeclampsia as a female-specific cardiovascular risk factor. However, evidence of integrated care concepts to optimize any future postpartum cardiovascular risk is scarce and recommendations by national guidelines are highly divergent. Both the German Association of Gynecology and Obstetrics (DGGG) and the Society of Obstetricians and Gynecologists of Canada (SOGC) advocate a medical check-up by a specialist for internal medicine or a nephrologist to exclude secondary causes of PE and to evaluate renal function, blood pressure, and other cardiovascular risk factors within the first months after delivery^14,15^. General check-ups and screening for cardiovascular risk factors should be repeated regularly according to the DGGG^14^ and the ESC^16^. In contrast, the National Institute for Health and Care Excellence (NICE) guidelines suggest carrying out a urine dipstick test 6—8 weeks postpartum and subsequent assessment of renal function if proteinuria persists^1^. Unfortunately, most physicians and most women are unaware of the associated long-term complications after PE, which highlights the need for multidisciplinary, coordinated health care programs after PE for primary and secondary prevention^17–19^. Currently, it remains unclear whether and to which extent specialized medical postpartum care is sought.

This large observational cohort study aimed to investigate the current postpartum care of women whose pregnancy was complicated by preeclampsia using claims data from German statutory health insurance. We analyzed postpartum utilization of primary and specialized care and antihypertensive medication prescriptions of patients with and without preeclampsia.

## Methods

### Study design and population

Women who had singleton live births between January 1, 2010, and December 31, 2017, were identified from statutory claims data of the AOK Baden-Wuerttemberg. The AOK Baden-Wuerttemberg is one of the major regional German statutory health insurance companies with 4.5 million insured persons. We analyzed a subsample of mothers whose data could be matched with the data of their child (222,779 women with 291,091 births) and excluded twin pregnancies (n = 5827), implausible dates of delivery (n = 964), implausible records of maternal age (n = 23), and those who were insured for less than 40% of the observation period (n = 26,365) from all analyses at baseline. Some patients met more than one exclusion criterion (see Supplementary Fig. S1). We did not exclude any preexisting cardiorenal complication at baseline as we aimed to evaluate postpartum medical care in general after PE. Follow-up data were collected until September 30, 2019. The final study cohort included 193,205 women with 258,344 births.

Pregnancies with a history of a preeclampsia were identified using ICD-10 codes (International Classification of Diseases) and current medication by ATC (Anatomical Therapeutic Chemical) classification systems (see Supplementary Table S1). Billing-relevant coding both in primary care and in the outpatient sector was used to identify exposure and outcome variables. The specialty of the health care provider was identified using the specialist group code of the lifelong physician’s number.

### Exposure variable

The study exposure variable involved having at least one pregnancy complicated by preeclampsia, which was defined as new onset of hypertension ≥ 140/90 mmHg after 20 weeks of gestation combined with at least one end-organ dysfunction, as defined by the DGGG based on coding of the International Classification of Diseases (ICD-10). ICD coding did not allow differentiating between mild or severe preeclampsia. All other patients served as the control group.

We considered births as the statistical subject for the current analyses. As one woman could contribute pregnancies and person-time to both unexposed and exposed groups during follow-up, we considered PE as a time-dependent variable. The “no PE” group included women (1) who never had a PE in any pregnancies, or (2) from the date of their first delivery without prior PE until the date of their first pregnancy with PE, irrespective of subsequent pregnancy outcomes. As an example, if a woman had two deliveries and only her second pregnancy was complicated by PE, she was considered “no PE” between her first and second delivery, but from then on was classified as “PE”.

### Outcome variables

Our primary outcome variables were postpartum utilization of primary and specialized care and prescription behavior for antihypertensive drugs after delivery. Among women’s health providers we included gynecologists, general practitioners, specialists for internal medicine, cardiologists, and nephrologists. We analyzed both the proportion of women who visited any doctor at certain time points and the mean rate of visits per quarter. Medical visits for any reason were registered. Similarly, the proportion of women receiving antihypertensive medication postpartum and the mean rates of the quarterly prescriptions of each antihypertensive drug was evaluated and compared among the two groups. Mean follow-up duration was 5.44 years. The follow-up period began with the date of delivery. End of follow-up included death, migration, change to another insurance company, or end of study period.

### Statistical analysis

Statistical analysis was performed using R version 4.0.2 and R-Studio v. 1.3.1056 for Windows (32/64 bit)^20^. The study cohort was stratified into women who experienced at least one PE during their pregnancies versus women without PE. Baseline characteristics were stated as absolute and relative counts or as means with standard deviation where appropriate.

The distribution of medical visits to the gynecologist, general practitioner, specialist for internal medicine, cardiologist, and nephrologist in the outpatient setting as well as the mean rates of quarterly prescriptions of antihypertensives were stratified by the occurrence of PE throughout the observation time after birth.

We used Pearson’s χ^2^ test with Yates’ continuity correction to compare the proportion of postpartum medical visits and antihypertensive drug medication among the women with and without PE. We used r-values to show the effect sizes (r < 0.1 = no effect, r < 0.3 = small effect, r < 0.5 = medium effect, and r > 0.5 = large effect).

Cox proportional hazard regression was calculated to estimate hazard ratios (HR) with 95% confidence intervals (CI) regarding the occurrence of time-dependent events (time between delivery and first antihypertensive prescription). Patients with preexisting antihypertensive medication were excluded. For our proportional hazard regression model, we stratified the patients according to the study exposure variable PE. The model was adjusted for confounders including maternal age, diabetes, and obesity that could plausibly affect the risk of requiring antihypertensive medication^21–23^. The HRs were interpreted as relative risks, e.g. a HR > 1 meant that the event-rate of the exposed group (PE) was > 1 times the event rate of the un-exposed group (no PE).

### Ethics approval

Ethical approval was obtained by the Ethics Committee of Tuebingen University Medical Faculty and University Hospital (380/2020BO) and granted an exemption from requiring informed consent as all data were sufficiently anonymized and cannot be traced to the study team. The study adheres to the STROBE guideline for observational cohort studies, including the RECORD extension, and all methods were performed in accordance with the Declaration of Helsinki.

## Results

### Baseline characteristics

The final study cohort was made up of 193,205 women with 258,344 singleton live births, including 12,060 women (6.24%) with at least one pregnancy complicated by PE. Baseline characteristics are shown in Table 1. As expected, women with PE had a higher prevalence of preexisting co-morbidities. New onset of adverse events developed more often after PE, for example, hypertension occurred in 31.37% of the women after PE compared to 7.83% after normotensive pregnancies.

**Table 1:** Clinical baseline characteristics of the study cohort.

Variable	Analysis set	PE	no PE
Women (%)	193,205 (100)	12,060 (6.2)	181,145 (93.8)
Births (%)	258,344 (100)	14,510 (5.6)	243,834 (94.4)
Age (years)	30.4 ± 5.4	30.7 ± 5.5	30.41 ± 5.4
*Medical history, n (%)*			
Obesity	36,674 (19)	4,318 (35.8)	32,356 (17.9)
Gestational diabetes	31,941 (16.5)	2,938 (24.9)	29,003 (16.)
Preterm birth (< 37 + 0)*	17,040 (6.6)	2,142 (14.8)	14,898 (6.1)
Small for gestational age*	7,689 (3)	528 (3.6)	7,161 (2.9)
Cardiovascular disease	4,533 (2.4)	343 (2.8)	4190 (2.3)
Hypertension	8,078 (4.2)	1175 (9.7)	6903 (3.8)
Cerebrovascular disease	1,144 (0.6)	93 (0.8)	1051 (0.6)
Chronic kidney disease	603 (0.3)	41 (0.3)	562 (0.3)
End-stage renal disease	45 (0.02)	3 (0.02)	42 (0.02)
Birth mode, n (%)*			
Cesarean section	26,561 (10.3)	1,991 (13.7)	24,570 (10.1)
Vaginal	57,443 (22.2)	2,344 (16.2)	55,099 (22.6)
Assisted vaginal delivery	4428 (1.7)	235 (1.6)	4,193 (1.7)
Unknown	170,289 (65.9)	10,317 (71.6)	159,972 (65.6)
*New onset adverse events, n (%)*^*†*^			
Cardiovascular disease^*†*^	4,436 (2.4)	366 (3.1)	4070 (2.3)
Hypertension^*†*^	17,058 (9.2)	3415 (31.47)	13,643 (7.8)
Cerebrovascular disease^*†*^	1,974 (1.0)	213 (1.8)	1761 (1)
Chronic kidney disease^*†*^	1,482 (0.8)	210 (1.8)	1272 (0.7)
End-stage renal disease^*†*^	161 (0.08)	31 (0.3)	130 (0.07)
Maternal death	194 (0.1)	14 (0.1)	180 (0.1)

Data are presented as mean ± standard deviation or absolute numbers (percentage). *Percentages refer to total number of births. ^*†*^Percentages refer to total number, excluding women with preexisting diagnosis of each entity.

### Outpatient postpartum medical visits

**Figure 1:**
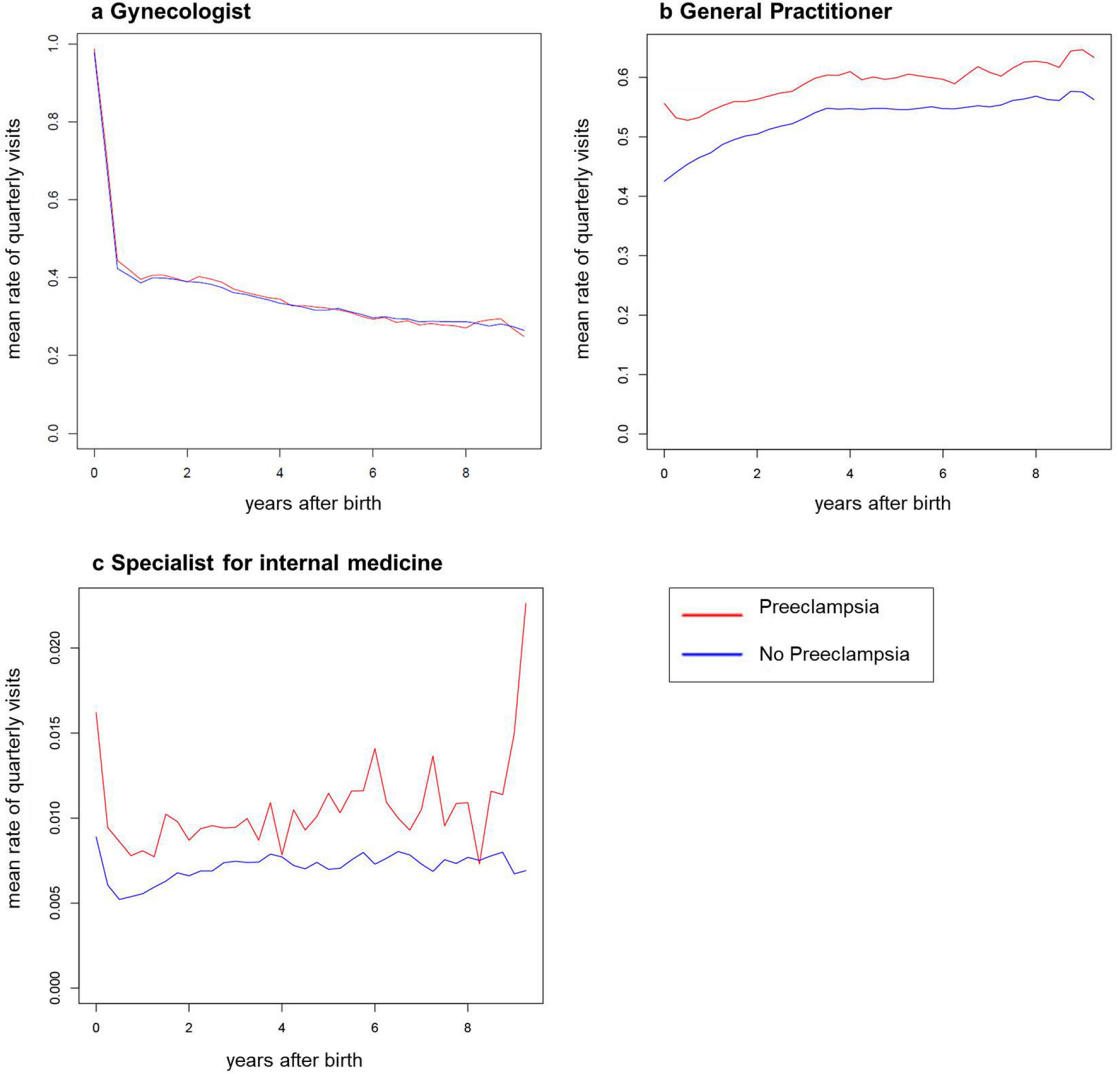
Postpartum care utilization in patients with and without PE. Mean rates of quarterly visits to the gynecologist (**a**), general practitioner (**b**), and specialist for internal medicine (**c**) in patients with (red) and without PE (blue) after delivery.

Investigating medical consultations according to quarterly medical visits, we found that most of the gynecological consultations took place during the first quarter after delivery, most likely mirroring standard postnatal follow-up. The rate of subsequent gynecological quarterly visits dropped sharply to 30–40%, while the general practitioner simultaneously became more important as the rate of quarterly visits rose up to 60% (Fig. 1). The rate of patients referred to specialists for internal medicine per quarter was low in both groups (< 2% in both groups).

**Figure 2:**
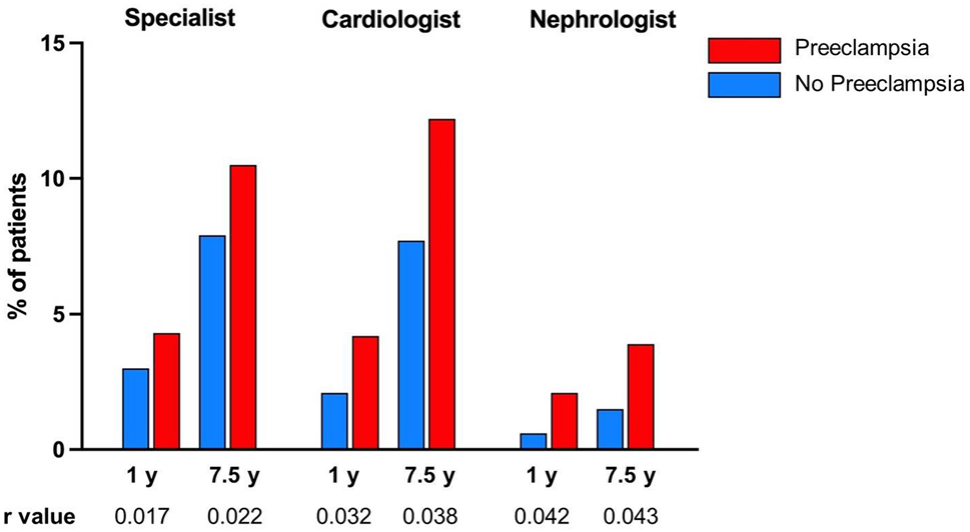
Relative number of patients who consulted a specialist for internal medicine, cardiologist, or nephrologist during follow-up stratified for the occurrence of PE. Data are presented as cumulative percentages of patients with (red) and without preeclampsia (blue) and were compared using χ^2^ test with Yates’ continuity correction; r values > 0.1 indicate a weak association. *P* values not shown, as for each *p* < 0.0001.

We then evaluated how many women with and without PE received specialized medical care after delivery as recommended by international guidelines^14,15^ and stratified the data according to medical profession. As shown in Fig. 2, only a minority of women visited specialists for internal medicine, cardiologists, or nephrologists during the first year after delivery (< 5% in all groups). During follow-up, the relative proportions of women who attended specialized outpatient care increased in both groups, presumably reflecting the onset of age-dependent comorbidities. By the end of follow-up, 10% of women after PE were referred at least once to a specialist for internal medicine, 12% to a cardiologist, and 4% to a nephrologist (Fig. 2). A full list of proportions at more time points can be found as Supplementary Table S2 online. Although specialized postpartum care was sought by more women after PE than by women without PE, the effect size indices revealed no considerable association between a history of PE and the utilization of specialized outpatient aftercare (Fig. 2). As an example, PE was not associated with more women being consulted by a nephrologist (2% vs. 0.6%, r = 0.042) nor by a cardiologist (4% vs. 2%, r = 0.032) during the first year after delivery.

### Antihypertensive drug prescriptions

**Figure 3:**
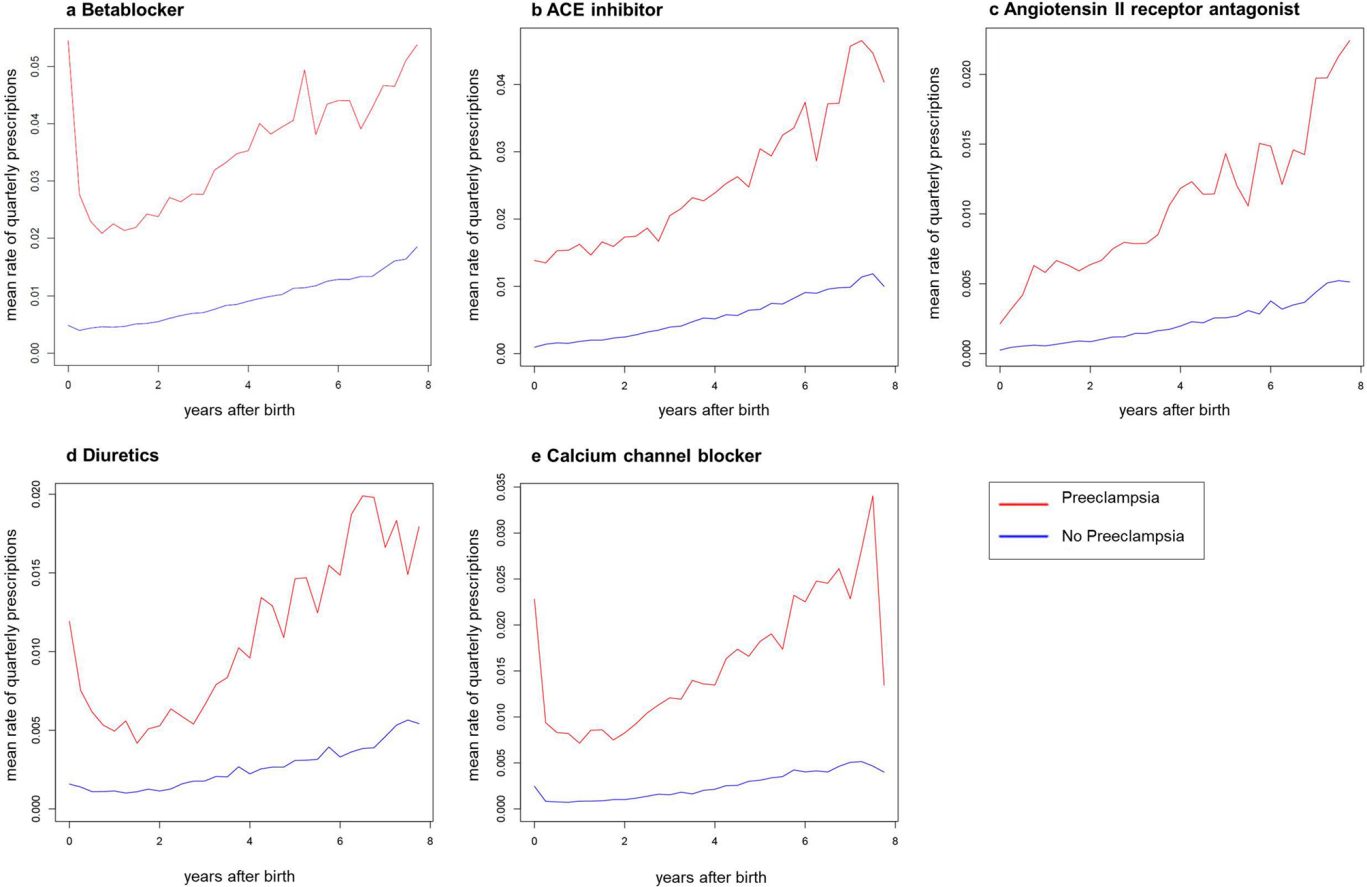
Prescription of antihypertensive medication in patients with and without PE. Mean rates of betablockers (**a**), ACE inhibitors (**b**), angiotensin II receptor antagonists (**c**), diuretics (**d**), and calcium channel blockers (e) prescribed quarterly and stratified for patients with (red) and without PE (blue) after delivery.

The number of women who needed any antihypertensive medication was substantially higher among the women after PE and increased over time (Table 2). ACE inhibitors and betablockers were the most commonly used antihypertensive drugs. Only few women required antihypertensive drug combinations (e.g., ACE inhibitor combinations 1.26% with vs. 0.12% without PE after 1 year). A history of PE was weakly associated with the intake of ACE inhibitors (e.g., r = 0.106 after 2 years) and betablockers (e.g., r = 0.118 after 1 year) at most time points after birth. As shown in Fig. 3, the quarterly prescriptions of any antihypertensives were higher among women after PE. This difference during the initial quarters after delivery most likely represents the higher prevalence of preexisting hypertension among preeclamptic women. During follow-up, the quarterly prescription rate among women with PE rapidly increased with a steeper slope compared to women without PE. Considering the high number of patients after PE using antihypertensive medications (Table 2) and the high incidence of new-onset hypertension after PE, the mean rates of quarterly prescriptions were low for each antihypertensive medication group. While 11.6% of the women with PE required betablockers after 7.5 years (Table 2), the maximum quarterly prescription rate of betablockers among those patients only reached 5.45% (Fig. 3).

**Table 2:** Number of patients receiving antihypertensive therapy during follow-up stratified for the occurrence of PE.

Antihypertensive therapy		No PE n = 243,834		With PE n = 14,510		r value
Betablocker	1y	3562	(1.5)	1210	(8.3)	**0.118**
	2y	4858	(2.0)	1368	(9.4)	**0.112**
	3y	5888	(2.4)	1485	(10.2)	**0.108**
	5y	7102	(2.9)	1626	(11.2)	**0.106**
	7.5y	7572	(3.1)	1679	(11.6)	**0.105**
ACE inhibitor	1y	1278	(0.5)	606	(4.2)	0.099
	2y	1927	(0.8)	799	(5.5)	**0.106**
	3y	2487	(1.0)	891	(6.1)	**0.104**
	5y	3246	(1.3)	1027	(7.1)	**0.104**
	7.5y	3639	(1.5)	1100	(7.6)	**0.104**
Angiotensin II receptor blocker	1y	370	(0.2)	179	(1.2)	0.054
	2y	574	(0.2)	239	(1.6)	0.058
	3y	756	(0.3)	283	(2.0)	0.06
	5y	1014	(0.4)	336	(2.3)	0.061
	7.5y	1151	(0.5)	374	(2.6)	0.063
Calcium channel blocker	1y	1364	(0.6)	526	(3.6)	0.083
	2y	1675	(0.7)	616	(4.2)	0.087
	3y	1992	(0.8)	684	(4.7)	0.089
	5y	2411	(1.0)	755	(5.2)	0.088
	7.5y	2645	(1.1)	799	(5.5)	0.089
Diuretics	1y	1340	(0.5)	370	(2.5)	0.057
	2y	1727	(0.7)	447	(3.1)	0.06
	3y	2111	(0.9)	515	(3.5)	0.062
	5y	2590	(1.1)	606	(4.2)	0.065
	7.5y	2825	(1.2)	646	(4.5)	0.066
ACE inhibitor combinations	1y	299	(0.12)	183	(1.26)	0.061
	2y	445	(0.18)	229	(1.58)	0.063
	3y	566	(0.23)	264	(1.82)	0.064
	5y	777	(0.32)	310	(2.14)	0.065
	7.5y	890	(0.37)	332	(2.29)	0.064
Angiotensin II receptor blocker combinations	1y	172	(0.07)	102	(0.70)	0.044
	2y	281	(0.12)	140	(0.96)	0.048
	3y	366	(0.15)	164	(1.13)	0.05
	5y	480	(0.20)	196	(1.35)	0.052
	7.5y	548	(0.22)	207	(1.43)	0.051

Data are presented as absolute numbers (cumulative percentages) and were compared using χ^2^ test with Yates’ continuity correction. Bold values indicate r values > 0.1. P values not shown, as for each row *p* < 0.0001. PE: preeclampsia; ACE: angiotensin-converting enzyme.

**Figure 4:**
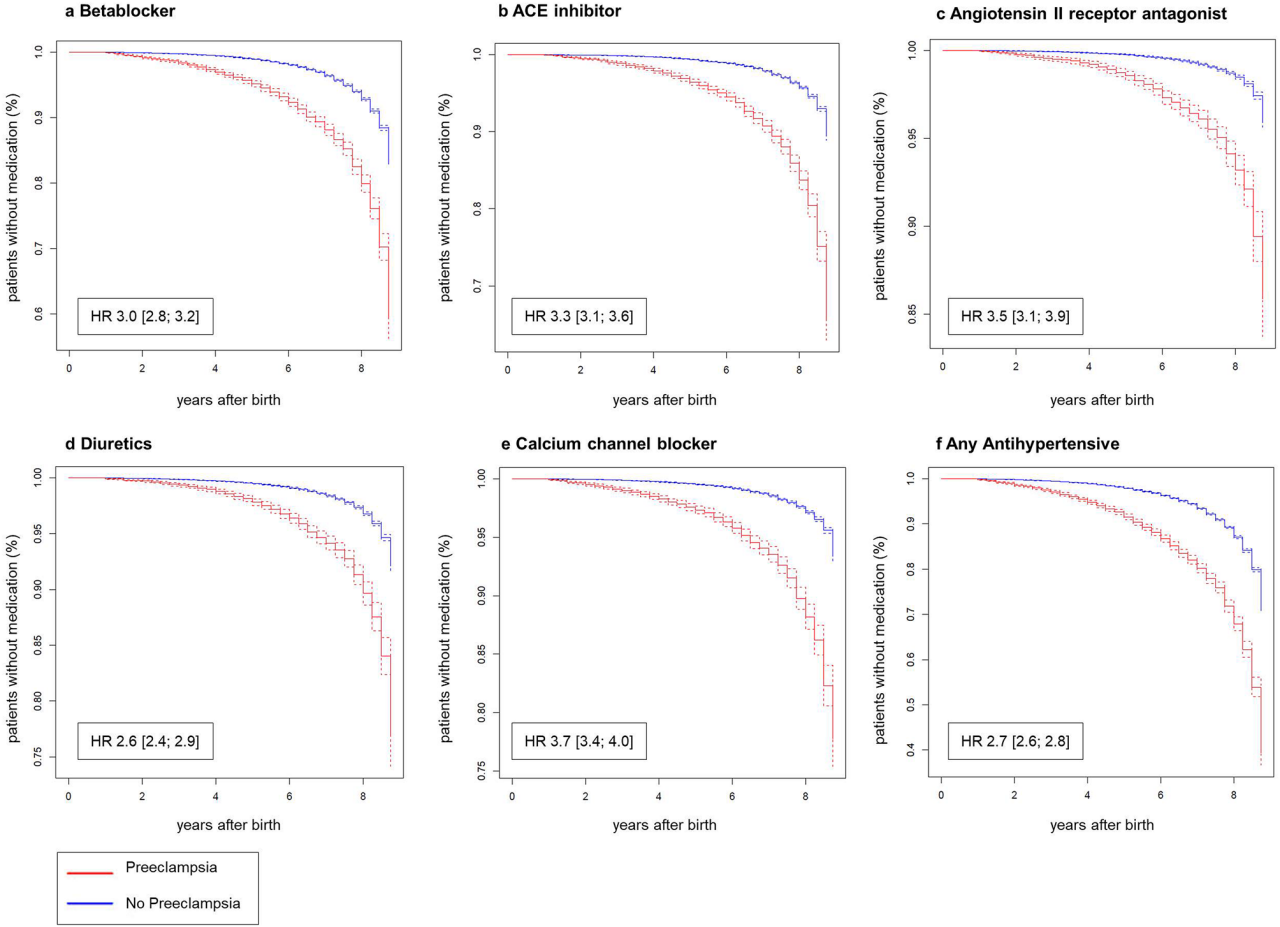
Cumulative hazard plots on the risk to take antihypertensive medication. Cumulative percentage of patients with (red) and without PE (blue) after delivery without betablockers (**a**), ACE inhibitors (**b**), angiotensin II receptor antagonists (**c**), diuretics (**d**), calcium channel blockers (**e**), and any antihypertensive medication (**f**). Hazard Ratios (HRs) and 95% CI were analyzed with the use of a Cox regression model adjusted for maternal age, diabetes, and obesity. Women with diagnosis of prior hypertension to were excluded. P values not shown, as for each *p* < 0.0001.

In Cox proportional regression analysis, women after PE were at a 2.7-fold increased risk of needing any antihypertensive medication after delivery compared to women without PE (HR 2.7 [2.6; 2.8]) as depicted in Fig. 4 and Table 3. Maternal age and diabetes seemed to have low effects (age HR 1.05 [1.05; 1.06]; diabetes HR 1.34 [1.28; 1.40].

**Table 3:** Cox proportional regression models onto first prescription of antihypertensive medications by risk exposures.

Outcome	Exposure	HR	95% CI
Betablockers	Preeclampsia	2.97	[2.78; 3.17]
	Maternal age	1.04	[1.04; 1.05]
	Diabetes	1.25	[1.18; 1.33]
	Obesity	1.52	[1.44; 1.61]
ACE inhibitors	Preeclampsia	3.30	[3.06; 3.55]
	Maternal age	1.10	[1.10; 1.11]
	Diabetes	1.61	[1.50; 1.72]
	Obesity	2.64	[2.48; 2.81]
Angiotensin II receptor antagonists	Preeclampsia	3.49	[3.10; 3.93]
	Maternal age	1.12	[1.11; 1.13]
	Diabetes	1.62	[1.46; 1.81]
	Obesity	2.81	[2.53; 3.12]
Diuretics	Preeclampsia	2.63	[2.40; 2.89]
	Maternal age	1.06	[1.06; 1.07]
	Diabetes	1.42	[1.31; 1.54]
	Obesity	3.08	[2.9; 3.32]
Calcium channel blockers	Preeclampsia	3.687	[3.37; 4.03]
	Maternal age	1.08	[1.07; 1.09]
	Diabetes	1.64	[1.51; 1.78]
	Obesity	1.97	[1.81; 2.13]
Any antihypertensive medication	Preeclampsia	2.68	[2.55; 2.81]
	Maternal age	1.05	[1.05; 1.06]
	Diabetes	1.34	[1.28; 1.40]
	Obesity	1.90	[1.82; 1.97]

Data are expressed as hazard ratios (HR) with 95% CI. Cox proportional regression modeling included age, diabetes, and obesity. Women with preexisting antihypertensive medication were excluded. P values not shown, as for each row *p* < 0.0001.

## Discussion

We investigated the postpartum medical care of women who experienced PE in a large retrospective database cohort of one of the major regional German statutory health insurance companies. We found that (i) only a minority of women after PE were referred to specialized outpatient care after delivery and that (ii) after PE, women took antihypertensive medication more frequently, and mean rates of quarterly prescriptions rapidly increased during the follow-up period.

According to our analyses of the mean rates of medical visits, gynecologists only have a short time frame after delivery to counsel and organize any necessary follow-up examinations for women after PE. Although international guidelines recommend a detailed examination by a specialist after PE to exclude secondary causes^14,15^, only a disproportionally small fraction of the women in our study was referred to any specialized outpatient care provider, reflecting the failure to match the increased long-term morbidity to be expected after PE^12,24^. The mere existence of guidelines does not appear to be effective enough and fails to be translated into clinical practice. Our analyses showed that both gynecologists and general practitioners represented the main health care providers for women after birth. Primary care and gynecological follow-up rates in our cohort were much higher than those reported by an US health insurance claims database, where only 58% of women presenting with hypertensive disorders in pregnancy were seen by any provider within 6 months after delivery^25^. The high follow-up rates with gynecologists and general practitioner in our analyses highlight a substantial missed opportunity to introduce integrated and interdisciplinary approaches to reduce the risk of short and long-term complications. Both gynecologists and general practitioners are capable of controlling potentially modifiable cardiovascular risk factors and advising their patients on adequate lifestyle modifications, such as quitting smoking, eating a healthy diet, maintaining a healthy body weight, and increasing general physical activity^1^. Unfortunately, several studies have revealed that knowledge of long-term risks and awareness of PE as a cardiovascular risk factor are limited among physicians^17–19,26^. Additionally, the mere documentation of an outpatient medical visit at the general practitioner or gynecologist does not necessarily guarantee adequate counseling on long-term sequelae. Educating women about their future cardiovascular risk and their risk of recurrence in following pregnancies comprise good clinical practice and are highlighted by several guidelines^1,14,15^. Patients have expressed a clear desire for intensified follow-ups with a specific focus on both the physical and psychological consequences of PE^19^. Secondary and tertiary prevention measures are usually hampered by the spontaneous recovery of maternal hypertension and proteinuria within a few weeks after delivery. Most patients hardly feel any symptoms and are distracted from their own health while navigating their new role from that of a pregnant woman to that of a mother caring for her infant. Only few interventional studies have been conducted on postpartum care. Riemer et al. recently demonstrated that early postpartum aerobic endurance exercise can reduce arterial stiffness, as a predictor for CVD^27^, and implementing a structured, bundled management of postpartum care after PE was shown to improve blood pressure control during the first 6 weeks after birth^28^.

Hypertension is a crucial risk factor for CVD that emerges after PE and accounts for substantially increased mortality^2^. Women after PE are diagnosed with chronic hypertension four times more often^24^ and 10–15 years earlier than women after a normotensive pregnancy^29^. After PE, up to 20% of the women remain hypertensive and one third show persisting proteinuria 6 months after delivery^30^, who should be referred for internal medicine or nephrology with further diagnostics according to guidelines^14,15^. Others even describe incidences of hypertension up to 41% 1 year after severe PE^31^. In line with these data, we observed new-onset hypertension among 31% of women after PE with similar proportions of women in need of any antihypertensive medication. In our cohort, hypertension developed early and increased rapidly during follow-up as mirrored by the rates of quarterly prescription of antihypertensive medication. Although prescription rates might not be the most accurate proxy for sequelae after PE or the quality of outpatient medical care, we presume a major discrepancy between the high incidence of persisting and new-onset hypertension following PE and insufficient referral to specialized outpatient care. During pregnancy as well as after delivery, regular self-measurement is essential for recognizing elevated blood pressure values at an early stage. The SNAP-HT trial recently demonstrated that monitoring postpartum hypertension remotely and titrating medication systematically improves blood pressure control among women after PE even after discontinuing antihypertensive medication^32^. In our study cohort, ACE inhibitors and betablockers were the most commonly prescribed antihypertensive drugs and the only antihypertensive drugs that were at least weakly associated with a history of PE. An advantage of renin-angiotensin-system blockers is that they are associated with a nephron-protective effect, reducing proteinuria and delaying end-stage renal disease^2^. However, ACE inhibitors and angiotensin-receptor blockers should be used with caution in women of childbearing age due to teratogenic effects^2^. Although betablockers have been questioned as a first-choice antihypertensive drug due to their adverse effects on lipid metabolism in general, betablockers were the most frequently prescribed drug in our cohort. Various intervention studies have investigated the benefit of giving furosemide to women postpartum after PE and found conflicting results, with either improved blood pressure at 7 days after delivery^33^ versus no improvement but less need for additional antihypertensive drugs^34^.

A limitation of our study is its retrospective design, rendering the results explorative rather than confirmatory. The major strength of our analysis is the large sample size and long follow-up period, reducing the potential sources of bias to a minimum. The quality of our data is guaranteed as far as possible by the use of medical records provided by a German insurance company and is prone to misclassification bias and missing data. In addition, certain information as the socioeconomic and smoking status that could potentially affect adherence to postpartum medical care is not well captured in claims-based datasets. Accessibility of medical data to researchers in Germany is restricted to claims data, as a comprehensive all-payer database is not available unlike in other countries^35,36^. We were able to integrate both inpatient and outpatient data in our analysis, thereby increasing quality and reliability of our data collection compared to other large studies based on self-report.

## Conclusions

Postpartum referral to specialized outpatient care after PE was insufficient, despite the early and rapidly increasing need for antihypertensive medication. Our findings highlight the major importance of gynecologists and general practitioners in postpartum outpatient care and emphasize the urgent need to implement a reasonable multidisciplinary follow-up strategy and prevention management in order to achieve long-term clinical benefits. Also, this work will stimulate future research to focus on evaluating the long-term benefit of early drug interventions and tools to accurately identify vulnerable patients prone to developing adverse outcomes.

## Supplementary Information

Supplementary Information.

## Data Availability

The data that support the findings of this study are available from AOK Baden-Wuerttemberg but restrictions apply to the availability of these data, which were used under license for the current study, and so are not publicly available. Data are however available from the authors upon reasonable request and with permission of AOK Baden-Wuerttemberg.

## Abbreviations

PE: Preeclampsia
ICD-10: International Classification of Diseases
ATC: Anatomical Therapeutic Chemical – Classification
CVD: Cardiovascular disease
ACE: Angiotensin converting enzyme
DGGG: German Society of Gynecology and Obstetrics
SOGC: Society of Obstetricians and Gynecologists of Canada
CKD: Chronic kidney disease
ESC: European Society of Cardiology
AOK: Allgemeine Ortskrankenkasse (General local health insurance)
CI: Confidence interval
