# Supplementary material for: Observational database study on preeclampsia and postpartum medical care up to 7.5 years after birth
Source: Sci Rep. 2022 Dec 8;12:21230. doi: 10.1038/s41598-022-25596-2 (PMC9732277; doi:10.1038/s41598-022-25596-2)
Supplement: Supplementary file 1 — Supplementary Information. [file 41598_2022_25596_MOESM1_ESM.docx]

**Observational database study on Preeclampsia and Postpartum medical care up to 7.5 years after birth**

Anna S. Scholz MD^1*^, Kathrin Hassdenteufel MD^1^, Raphael Gutsfeld^2^, Mitho Müller^2^, Maren Goetz MD^3^, Armin Bauer PhD^4^, Markus Wallwiener MD^1^, Sara Y. Brucker MD^4^, Stefanie Joos MD^5^, Miriam Giovanna Colombo MPH^5^, Sabine Hawighorst‑Knapstein MD^6^, Ariane Chaudhuri MD^6^, Frauke Beck^6^, Stephanie Wallwiener MD^1^

^1^ Department of Gynecology and Obstetrics, Heidelberg University Hospital, Heidelberg, Germany

^2^ Department of Psychology, Ludwig Maximilian University, Munich, Germany

^3^ Department of General Pediatrics, University Children’s Hospital, Heidelberg, Germany

^4^ Department of Women’s Health, University Hospital Tuebingen, Tuebingen, Germany

^5^ Institute for General Practice and Interprofessional Care, University Hospital Tuebingen, Tuebingen, Germany

^6^ Department of Health Promotion, AOK Baden-Wuerttemberg, Stuttgart, Germany

**Supplemental Figure S1 Patient flow chart.**

**
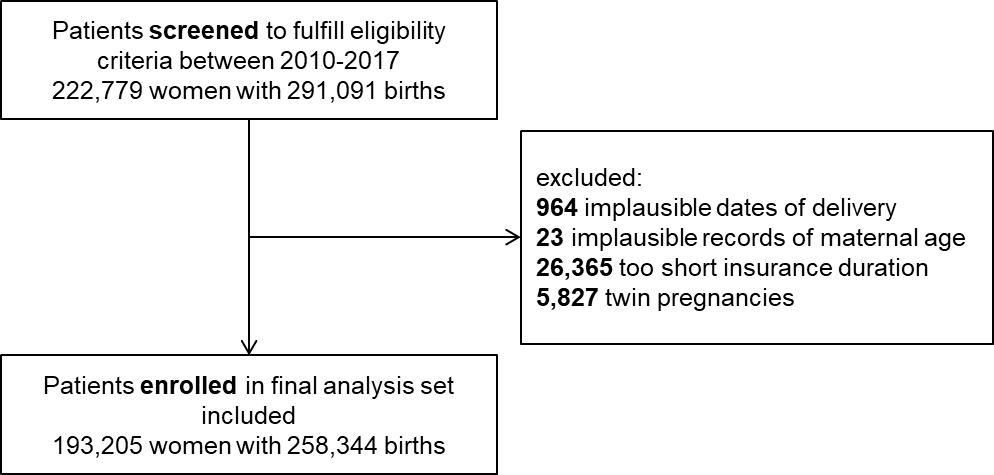
**

**Supplemental Table S1 List of ICD-10, ATC, and specialist group codes for definitions of exposure and outcome variables.**

|  | **ICD-10, ATC, and specialist group codes** |
| --- | --- |
| Preeclampsia | O14.- |
| Hypertension | I10.-, I11.-, I12.-, I13.-, I15.- |
| CVD | I20.-, I21, I22.-, I23.-, I24.-, I25.-, I51.6, I51.8; I51.9 |
| Chronic kidney disease | N18.-, N19, Z49.1, Z49.2, Z94.0, Z99.2 |
| End-stage renal disease | N18.4, N18.5, Z49.1, Z49.2, Z94.0, Z99.2 |
| Cerebrovascular disease | G45.-, I60.-, I61.-, I62.-, I63.-, I64.-, I65.-, I66.-, I67.-, I69.- |
| Preterm birth | 060.1, 060.3, P07.2, P07.3 |
| Obesity | E66.- E78.- |
| Mode of delivery: |  |
| Vaginal | O80 |
| Assisted vaginal delivery | O81 |
| Cesarean section | O82 |
| Medication: |  |
| Betablocker | C07.- |
| ACE inhibitor | C09A.-, C09B |
| Angiotensin II receptor antagonist | C09C.-, C09D |
| Calcium channel blocker | C08.- |
| Diuretics | C03.- |
| Medical visits: |  |
| General practitioner | 1-3 |
| Gynecologist | 15-18 |
| Specialist for internal medicine | 23 |
| Cardiologist | 28 |
| Nephrologist | 29 |

ICD-10: International Classification of Diseases; ATC: Anatomical Therapeutic Chemical – Classification. Specialist group codes belong to the lifelong physician’s number.

**Supplemental Table S2 Number of patients who consulted a gynecologist, general practitioner, specialist, cardiologist, or nephrologist during follow-up stratified for the occurrence of preeclampsia.**

| Type of medical care utilization | | No PE  n=243,834 | | With PE  n=14,510 | | p value | r value |
| --- | --- | --- | --- | --- | --- | --- | --- |
| Gynecologist | 1y | 242,468 | (99.4) | 14,465 | (99.7) | 0.006 | 0.008 |
|  | 2y | 242,828 | (99.6) | 14,482 | (99.8) | 0.004 | 0.008 |
|  | 3y | 242,992 | (99.7) | 14,485 | (99.8) | 0.04 | 0.007 |
|  | 5y | 243,168 | (99.7) | 14,489 | (99.9) | 0.298 | 0.006 |
|  | 7.5y | 243,227 | (99.8) | 14,489 | (99.9) | 1 | 0.005 |
| General practitioner | 1y | 214,117 | (87.8) | 13,244 | (91.3) | <0.0001 | 0.025 |
|  | 2y | 230,362 | (94.5) | 13,943 | (96.1) | <0.0001 | 0.016 |
|  | 3y | 235,216 | (96.5) | 14,156 | (97.6) | <0.0001 | 0.014 |
|  | 5y | 237,601 | (97.4) | 14,250 | (98.2) | <0.0001 | 0.011 |
|  | 7.5y | 238,014 | (97.6) | 14,273 | (98.4) | <0.0001 | 0.011 |
| Specialist for internal medicine  | 1y | 7,355 | (3.0) | 625 | (4.3) | <0.0001 | 0.017 |
|  | 2y | 11,407 | (4.7) | 919 | (6.3) | <0.0001 | 0.018 |
|  | 3y | 14,391 | (5.9) | 1,128 | (7.8) | <0.0001 | 0.018 |
|  | 5y | 17,741 | (7.3) | 1,395 | (9.6) | <0.0001 | 0.021 |
|  | 7.5y | 19,381 | (7.9) | 1,528 | (10.5) | <0.0001 | 0.022 |
| Cardiologist | 1y | 5,237 | (2.1) | 609 | (4.2) | <0.0001 | 0.032 |
|  | 2y | 9,416 | (3.9) | 974 | (6.7) | <0.0001 | 0.033 |
|  | 3y | 12,744 | (5.2) | 1,250 | (8.6) | <0.0001 | 0.034 |
|  | 5y | 16,783 | (6.9) | 1,609 | (11.1) | <0.0001 | 0.038 |
|  | 7.5y | 18,845 | (7.7) | 1,764 | (12.2) | <0.0001 | 0.038 |
| Nephrologist | 1y | 1,485 | (0.6) | 309 | (2.1) | <0.0001 | 0.042 |
|  | 2y | 2,201 | (0.9) | 394 | (2.7) | <0.0001 | 0.042 |
|  | 3y | 2,727 | (1.1) | 454 | (3.1) | <0.0001 | 0.042 |
|  | 5y | 3,344 | (1.4) | 523 | (3.6) | <0.0001 | 0.042 |
|  | 7.5y | 3,663 | (1.5) | 560 | (3.9) | <0.0001 | 0.043 |

Data are presented as absolute numbers (percentages) and were compared using χ^2^ test with Yates’ continuity correction; r values >0.1 indicate a weak association. PE: preeclampsia
